# The Prevalence of COVID-19 in Europe by the End of November 2022: A Cross-Sectional Study

**DOI:** 10.7759/cureus.33546

**Published:** 2023-01-09

**Authors:** Ahmad A Alrasheedi

**Affiliations:** 1 Department of Family and Community Medicine, Qassim University, Buraidah, SAU

**Keywords:** median age, vaccine, testing, european union, europe, covid-19, coronavirus, case-fatality

## Abstract

Background

The world has been affected differently by the coronavirus disease 2019 (COVID-19), and Europe reaped the largest number of cases and deaths. Moreover, COVID-19 statistics are dynamic.

Objectives

The current study aimed to use COVID-19 data to examine the COVID-19 prevalence in Europe by the end of November 2022 and compare the findings globally.

Methods

The primary data on COVID-19 for each European country were obtained from the “Worldometer” website. The data include the cumulative incidence of COVID-19 per country, the cumulative number of deaths, the total number of tests performed, the number of cases per million population, the number of deaths per million, the number of tests per million, and the total population. The case-fatality rate was calculated (number of deaths/number of cases). In addition, the median age and the vaccination coverage rate (people who received two doses) for each European country were extracted from the “United Nations” website and the “Our World in Data” website, respectively. To compare European countries to the globe, COVID-19 data for each continent were obtained. The analysis of variance (ANOVA) test was used to compare variances across the means of the four parts of Europe based on the geographic division. An independent sample t-test was also used to compare the means between the European Union (EU) states and non-EU states. The Spearman correlation coefficient was used to determine the relationship between different variables across Europe.

Results

As of December 1, 2022, about 648 million COVID-19 cases and 6.6 million deaths have been recorded worldwide. Europe accounted for nearly 36.8% and 29.5% of all cases and deaths, respectively. Based on the number of deaths per million, Europe was the most affected continent after South America. Nearly 6.8 billion tests have been conducted worldwide, 41% done in Europe; 43 European countries have performed tests more than their population. However, COVID-19 statistics were inconsistent across the four parts of Europe. A significant difference was noticed between Eastern Europe and others, especially Northern Europe and Western Europe. By affiliation with the EU, there was no significant difference. For global comparison, the mean deaths per million, the mean cases per million, and the mean tests per population for European countries were higher than those of the world’s countries, although they recorded a lower mean case-fatality rate (CFR). Thirteen European countries were among the 15 most affected countries worldwide based on the number of deaths per million, most located in Eastern and Southern Europe. The number of cases and the number of deaths were significantly proportional to the number of tests performed.

Conclusions

By the end of November 2022, Europe had the most cases of COVID-19, the most deaths, and the most tests performed, even though it accounts for 9.4% of the world’s population. However, COVID-19 data were inconsistent across the four parts of Europe, especially between Eastern Europe and others. Given the natural immunity acquired during the three years and the excellent vaccine coverage in Europe, it is essential to reconsider the definition of a suspected case and establish more specific criteria for testing.

## Introduction

In December 2019, a novel coronavirus, severe acute respiratory syndrome coronavirus 2 (SARS-CoV-2), causing coronavirus disease 2019 (COVID-19), emerged, and it has posed a global health threat, causing an ongoing pandemic over the world [1]. The spectrum of COVID-19 symptoms is broad. Most symptomatic patients present with respiratory tract symptoms, and most patients experience mild illness [1,2]. The World Health Organization (WHO) developed a case definition for COVID-19 [3]. Based on the last update, a suspected case of SARS-CoV-2 infection includes a person who meets clinical or epidemiological criteria. The clinical criteria include acute onset of fever and cough; or acute onset of three or more of the following signs or symptoms: fever, cough, general weakness/fatigue, headache, myalgia, sore throat, coryza, dyspnea, nausea/diarrhea/anorexia. The epidemiological criteria include a history of contact with a probable (a patient who meets both the clinical and epidemiological criteria) or confirmed case. However, because of the high rate of asymptomatic SARS-CoV-2 infections, a confirmed case of COVID-19 does not require the criteria for a suspect case to be met, but simply positive a nucleic acid amplification test (most commonly a reverse-transcription polymerase chain reaction (RT-PCR) assay) [3,4]. On the other hand, COVID-19 can also be diagnosed using SARS-CoV-2 antigen-detection rapid diagnostic tests (less accurate) in persons who meet the criteria for a suspected case [3].

In the beginning, China experienced the majority of the burden associated with COVID-19 in the form of morbidity and mortality, but over time, specifically from mid-February, the COVID-19 menace moved to Europe, particularly Italy and Spain [5]. At the same time, cases started to appear in other parts of the world, as the “Worldometer” data indicate [6]. So, on March 11, 2020, the WHO characterized COVID-19 as a pandemic [1].

Over approximately three years, the world has suffered from the pandemic unevenly. COVID-19 statistics have been inconsistent across the globe and may change dramatically over time [7]. In contrast to initial expectations, Africa was the continent least affected by the pandemic [7]. By the end of September 2022, Europe was the most affected continent based on the number of deaths and cases [7]. While the continent of Asia, where the pandemic originated, was less affected, taking into account the huge continent’s population compared to Europe [7]. Countries vary widely in their COVID-19 statistics. The rationale for these differences may be related to each country’s resources, testing strategies, politics, health insurance systems, reporting methods, and other unknown factors [7,8]. Additionally, demographic characteristics, such as the median age difference, influence the COVID-19 pandemic’s course, as infections with SARS-CoV-2 cause more significant mortality in the elderly [5,7]. Europe has the highest median age [7,9]. Furthermore, the difference in vaccination coverage is another crucial factor [10]. However, despite the deployment of COVID-19 vaccines, Europe became the pandemic’s epicenter again in late 2021 [11].

Europe covers about 10 million km^2^ (6.8% of land area), making it the second-smallest continent. Europe has about 10% of the world’s population [12]. For statistical convenience, the United Nations divided Europe into four regions: Eastern Europe, Northern Europe, Southern Europe, and Western Europe [13]. Further European integration by some states led to forming the European Union (EU), which consists of 27 states [12]. Given its size and diversity, Europe varies significantly across and within its regions regarding cultures, economics, government systems, and healthcare services [14].

Understanding the epidemiology of COVID-19 in the world and in each continent helps researchers and decision-makers explore the best ways to deal with COVID-19 and may provide lessons for a more effective response to public health emergencies. The world has been affected differently by this pandemic, and the continent of Europe reaped the largest number of cases and deaths. It seems that there is a significant difference across Europe, as in the continents of Asia and Africa [7,15]. Moreover, understanding of COVID-19 is still evolving, and COVID-19 statistics are dynamic. So, the current study aimed to use COVID-19 statistics to examine the prevalence of COVID-19 in Europe by the end of November 2022 and compare it to the global one.

## Materials and methods

Unless otherwise specified, the primary data on COVID-19 were obtained from the “Worldometer” website [6]. The data on COVID-19 for all world countries/territories were copied at the end of 2020, 2021, and November 2022 [6]. Then, they were stored in Excel files. The data used in this analysis consists of the cumulative incidence of COVID-19 (confirmed cases) per country, the cumulative number of deaths, the total number of tests performed, the total number of cases per million population, the total number of deaths per million population (D/M), the total number of tests per million population, and the total population. Data from cruise ships were excluded.

According to the objectives, the required data for each European country were obtained from the stored files. In addition, each European country’s median age for the year 2021 was extracted from the United Nations website [9]. The rate of vaccination coverage (people with a complete initial protocol: two doses) was also extracted from the “Our World in Data” website [10]. The case-fatality rate (CFR) was calculated by dividing the number of deaths by the number of confirmed cases. A CFR is generally expressed as a percentage [16].

To better compare COVID-19 statistics across the continent, European countries were classified into four groups based on their geographic location. The current study adopted the United Nations classification [13]. In another way, European countries were also analyzed based on their affiliation with the EU. Results were presented as numbers, percentages, and means with standard deviation (SD) as appropriate. Additionally, to evaluate the impact of the pandemic on Europe to the globe, COVID-19 data for each continent were obtained. The mean CFR, mean D/M, and mean cases per million for all world countries/territories and per continent were also measured. To avoid using too many digits, the number of tests per population is calculated, instead of using the number of tests per million, by dividing the number of tests by the population.

Statistical analysis was carried out using Statistical Package for Social Sciences (SPSS, version 26). The analysis of variance (ANOVA) test was used to compare variances across the means of the four parts of Europe based on the geographic division. If there are statistically significant differences between the groups as a whole, the Tukey post hoc test will be used to show which groups differed from each other. An independent sample t-test was also used to compare the means between the EU states and non-EU states. The Spearman correlation coefficient was used to determine the relationship between different variables across Europe. A P value of less than 0.05 was considered significant. Ethical approval from an Institutional Review Board was not required due to the secondary analysis of publicly available data.

## Results

By the end of November 2022, Europe was the continent with the most cases of COVID-19, the most deaths, and the most tests performed, even though it accounts for 9.4% of the world’s population, as shown in Table 1. Specifically, Europe has recorded nearly 238 million confirmed COVID-19 cases and about 2 million deaths, 36.82% and 29.54% of all cases and deaths, respectively. Based on the number of deaths per million, Europe was the most affected continent after South America, while Africa was the least affected. Among continents, the CFR ranged from 0.17% to 2.05%.

**Table 1:** COVID-19 statistics among the six continents by the end of November 2022 (sorted according to the number of deaths per million). *North America includes Mexico and Caribbean countries. ^#^CFR: case-fatality rate; C/M: the number of cases per million; D/M: the number of deaths per million; SD: standard deviation. ^¥^The mean is the sum of all values in the data set divided by the total number of values. While the CFR (not mean CFR), for example, is the total number of deaths of all countries included/the total number of cases of the same countries *100.

	South America	Europe	North America*	Oceania	Asia	Africa	All
No. of cases	65,136,648 (10.06%)	238,535,825 (36.82%)	119,184,914 (18.40%)	12,998,302 (2.01%)	199,231,454 (30.76%)	12,709,355 (1.96%)	647,796,498
No. of deaths	1,335,850 (20.12%)	1,961,162 (29.54%)	1,564,392 (23.56%)	22,239 (0.33%)	1,498,223 (22.56%)	258,080 (3.89%)	6,639,946
CFR^#^	2.05%	0.82%	1.31%	0.17%	0.75%	2.03%	1.03%
Mean CFR^¥^ ±SD^#^	1.86% ±1.32	0.91% ±0.84	1.09 ±0.90	0.37% ±0.44	1.39% ±2.63	1.78% ±1.31	1.31% ±1.68
C/M^#^	148,818	319,093	199,259	299,024	42,287	9,035	81,536
Mean C/M ±SD	178,896 ±135,476	378,408 ±176,374	220,976 ±162,665	193,907 ±143,523	145,298 ±161,171	42,127 ±103,296	187,003 ±188,560
D/M^#^	3,052	2,623	2,615	512	318	183	836
Mean D/M ±SD	2,330 ±1,545	2,583 ±1,235	1,464 ±841	433 ±555	666 ±758	323 ±522	1,189 ±1,259
No. of tests	239,367,290 (3.5%)	2,795,462,875 (40.88%)	1,274,984,196 (18.64%)	88,290,425 (1.29%)	2,330,946,763 (34.09%)	109,327,080 (1.60%)	6,838,378,629
Tests/pop.	0.55	3.74	2.13	2.03	0.49	0.08	0.86
Mean test/ pop. ±SD	0.98 ±0.85	4.38 ±5.15	2.61 ±3.39	1.56 ±1.52	1.91 ±3.34	0.21 ±0.31	2.06 ±3.54
Population	437,694,443	747,543,837	598,140,916	43,469,030	4,711,356,783	1,406,728,744	7,944,933,753

Given the geographical division of Europe, Western Europe had the most cases per million but the fewest deaths per million and the lowest CFR. In contrast, Eastern Europe had the highest CFR and was the most affected based on the number of deaths per million. Northern Europe performed the largest number of tests relative to the population, in contrast to Eastern Europe (Table 2). Between the EU states and non-EU states, there was no significant difference in COVID-19 statistics in the number of deaths per million. However, the EU countries reported more cases per million, performed more tests per population, and had a lower CFR value. Cyprus is a member of the EU but not included in the analysis because it is a part of Asia, according to the “Worldometer” website and the United Nations [13].

**Table 2:** COVID-19 statistics for Europe by geographical division and by affiliation to the European Union at the end of November 2022. *EU: The European Union; CFR: case-fatality rate; C/M: the number of cases per million; D/M: the number of deaths per million.

	By geographical division				By affiliation to the EU*	
	Eastern Europe (N=10)	Northern Europe (N=13)	Southern Europe (N=16)	Western Europe (N=9)	EU states (N=26)	Non-EU states (N=22)
No. of cases	48,033,583 (20.14%)	37,539,028 (15.74%)	55,261,869 (23.17%)	97,701,345 (40.96%)	175,946,550 (73.76%)	62,589,275 (26.24%)
No. of deaths	856,079 (43.65%)	263,956 (13.46%)	431,609 (22.01%)	409,518 (20.88%)	1,173,510 (59.84%)	787,652 (40.16%)
CFR*	1.78%	0.70%	0.78%	0.42%	0.67%	1.26%
C/M*	164,576	350,414	364,417	496,176	396,314	206,167
D/M*	2,933	2,464	2,846	2,080	2,643	2,594
No. of tests	472,607,801 (16.91%)	731,881,485 (26.18%)	904,805,597 (32.37%)	686,167,992 (24.55%)	1,890,580,448 (67.63%)	904,882,427 (32.37%)
Tests/pop.	1.62	6.83	5.97	3.48	4.26	2.98
Population	291,862,993 (39.04%)	107,127,633 (14.33%)	151,644,484 (20.29%)	196,908,727 (26.34%)	443,957,880 (59.39%)	303,585,957 (40.61%)

Statistically, COVID-19 data were inconsistent across the four parts of Europe regarding the mean CFR, the mean cases per million, the mean deaths per million, and the mean vaccination coverage rate, as shown in Table 3. Northern Europe was the least affected based on the mean deaths per million. Western Europe had the highest mean vaccination coverage rate, while Eastern Europe had the lowest. The post hoc analysis revealed a significant difference in the variance of means between Eastern Europe and others, especially Northern Europe and Western Europe (Table 4). Also, there was a significant difference in the mean D/M between Northern Europe and Southern Europe. In contrast, there was no significant difference in the variance of means between the EU states and non-EU states.

**Table 3:** Comparison of variance of means across Europe by geographical division and by affiliation to the European Union by the end of November 2022. *EU: The European Union, SD: Standard deviation, CFR: case-fatality rate, C/M: the number of cases per million, D/M: the number of deaths per million, Vac: vaccination coverage rate (the percentage of people who complete the initial protocol). ^#^Analysis of variance (ANOVA) test: A P value of <0.05 is considered significant. ^¥^Independent-samples t-test: A P value of <0.05 is considered significant.

	By geographical division					By affiliation to the EU*		
	Eastern Europe (N=10)	Northern Europe (N=13)	Southern Europe (N=16)	Western Europe (N=9)	P value^#^	EU states (N=26)	Non-EU states (N=22)	P value^¥^
Mean median Age ±SD*	40.9 ±2.31	40.8 ±2.79	42.8 ±2.93	43.4 ±4.51	0.197	42.3 ±2.17	41.3 ±4.14	0.279
Mean CFR* ±SD	1.77% ±0.68	0.44% ±0.25	1.02% ±1.01	0.42% ±0.12	0.000	0.88% ±0.68	0.94% ±1.01	0.823
Mean C/M* ±SD	204,488 ±101,364	442,218 ±142,133	362,628 ±201,825	489,761 ±77,969	0.001	393,356 ±139,135	358,378 ±214,011	0.491
Mean D/M* ±SD	3,393 ±1,342	1,739 ±982	3,119 ±1,114	2,006 ±485	0.001	2,826 ±1,122	2,280 ±1,328	0.134
Mean Tests/pop. ±SD	1.66 ±1.31	6.11 ±6.15	4.22 ±4.32	5.19 ±6.65	0.207	4.86 ±5.66	3.81 ±4.41	0.481
Mean Vac.* ±SD	49.44% ±13.56	74.90% ±5.28	66.43% ±25.28	72.81% ±4.55	0.004	69.05% ±14.32	62.50% ±22.45	0.235

**Table 4:** Multiple comparisons of variance of means between the four parts of Europe by geographical division using the Tukey post hoc test. *The mean difference is significant at the 0.05 level. ^#^The mean median age and the mean test/population were not included in the table because the ANOVA test (Table 3) did not show any significant difference.

Dependent Variable^#^	(I) Based on geographic location	(J) Based on geographic location	Mean Difference (I-J)	Sig.	95% Confidence Interval	
					Lower Bound	Upper Bound
Case-fatality rate	Eastern Europe	Northern Europe	1.33554^*^	0.000	0.5728	2.0983
		Southern Europe	.75275^*^	0.041	0.0217	1.4838
		Western Europe	1.35733^*^	0.000	0.5241	2.1906
	Northern Europe	Southern Europe	-0.58279-	0.114	-1.2599-	.0943
		Western Europe	0.02179	1.000	-0.7646-	.8082
	Southern Europe	Western Europe	0.60458	0.158	-0.1510-	1.3602
Cases per million	Eastern Europe	Northern Europe	-237727.977-^*^	0.003	-406572.31-	-68883.64-
		Southern Europe	-168140.275-^*^	0.039	-329956.08-	-6324.47-
		Western Europe	-285273.344-^*^	0.001	-469711.09-	-100835.60-
	Northern Europe	Southern Europe	69587.702	0.605	-80298.48-	219473.88
		Western Europe	-47545.368-	0.885	-221610.85-	126520.12
	Southern Europe	Western Europe	-117133.069-	0.256	-284389.57-	50123.43
Deaths per million	Eastern Europe	Northern Europe	1653.423^*^	0.003	477.82	2829.03
		Southern Europe	273.100	0.919	-867.92-	1414.12
		Western Europe	1386.500^*^	0.030	102.32	2670.68
	Northern Europe	Southern Europe	-1380.323-^*^	0.006	-2439.41-	-321.24-
		Western Europe	-266.923-	0.935	-1478.88-	945.03
	Southern Europe	Western Europe	1113.400	0.070	-65.04-	2291.84
Vaccination coverage	Eastern Europe	Northern Europe	-25.45650-^*^	0.004	-44.0528-	-6.8602-
		Southern Europe	-16.98800-	0.065	-34.7189-	0.7429
		Western Europe	-23.36622-^*^	0.016	-43.3217-	-3.4108-
	Northern Europe	Southern Europe	8.46850	0.539	-8.3525-	25.2895
		Western Europe	2.09028	0.991	-17.0613-	21.2418
	Southern Europe	Western Europe	-6.37822-	0.788	-24.6906-	11.9342

Table 5 shows COVID-19 statistics in Europe by country. Of the 15 worst European countries, 13 were from Eastern Europe and Southern Europe. Globally, Austria was the most testing country relative to its population (22 times/population), followed by Denmark. In contrast, Bosnia and Herzegovina was the least testing country, although it has tested the equivalent of just over half its population. Additionally, the lowest vaccination coverage rate was in Bosnia and Herzegovina (26.17%), followed by Bulgaria (30.58%).

**Table 5:** COVID-19 statistics for Europe by country by the end of November 2022 (sorted according to the number of deaths per million). *Affiliation of the country based on the geographic division: E: Eastern Europe, N: Northern Europe, S: Southern Europe, W: Western Europe. Additionally, the “+” sign indicates that the country belongs to the European Union. ^#^CFR: case-fatality rate; D/M: the number of deaths per million; age: median age in years; Vac.: vaccination coverage rate (the percentage of people who complete the initial protocol).

	Aff.*	No. of cases	No. of deaths	CFR^#^	D/M^#^	No. of tests	Age^#^	Vac.^#^ (%)	Population
Bulgaria	E+	1,287,256	38,039	2.96	5,558	10,820,606	44.5	30.58	6,844,597
Hungary	E+	2,166,352	48,287	2.23	5,027	11,394,556	42.7	62.27	9,606,259
Bosnia and Herzegovina	S	400,548	16,202	4.04	4,986	1,879,420	41.8	26.17	3,249,317
North Macedonia	S	344,710	9,568	2.78	4,597	2,141,032	38.8	40.03	2,081,304
Montenegro	S	283,719	2,790	0.98	4,443	2,664,018	38.2	45.43	627,950
Croatia	S+	1,253,761	17,316	1.38	4,266	5,362,362	43.7	55.83	4,059,286
Czechia	E+	4,558,202	41,882	0.92	3,901	56,615,622	42.6	65.67	10,736,784
Slovakia	E+	1,855,953	20,733	1.12	3,797	7,354,436	40.6	45.68	5,460,193
Lithuania	N+	1,276,076	9,434	0.74	3,544	10,258,663	43.7	68.35	2,661,708
Romania	E+	3,296,834	67,276	2.04	3,535	25,759,760	41.9	41.28	19,031,335
San Marino	S	22,167	119	0.54	3,491	157,634	46.3	70.15	34,085
Slovenia	S+	1,258,446	6,932	0.55	3,336	2,785,139	43.2	57.66	2,078,034
Greece	S+	5,360,506	34,178	0.64	3,313	99,637,289	44.7	73.59	10,316,637
Latvia	N+	961,627	6,086	0.63	3,292	7,746,372	43.6	70.57	1,848,837
Gibraltar	S	20,184	110	0.54	3,264	534,283	41.7	126.76	33,704
Poland	E+	6,352,755	118,319	1.86	3,135	37,796,070	40.9	56.7	37,739,785
Italy	S+	24,260,660	181,098	0.75	3,005	257,057,363	46.8	81.26	60,262,770
Moldova	E	595,073	11,918	2	2,970	3,216,305	36.1	32.79	4,013,171
United Kingdom	N	24,000,101	196,821	0.82	2,873	522,526,476	39.6	74.63	68,497,907
Belgium	W+	4,636,264	33,057	0.71	2,833	36,111,052	40.9	78.63	11,668,278
Russia	E	21,590,828	392,002	1.82	2,689	273,400,000	38.8	54.24	145,805,947
Ukraine	E	5,336,293	110,505	2.07	2,558	32,603,805	40.8	38.17	43,192,122
Portugal	S+	5,542,265	25,450	0.46	2,510	45,641,725	45	86.46	10,140,570
Spain	S+	13,595,504	115,901	0.85	2,481	471,036,328	43.9	85.49	46,719,142
France	W+	37,846,799	158,950	0.42	2,424	271,490,188	41.6	78.32	65,584,518
Austria	W+	5,561,633	21,210	0.38	2,339	202,723,557	42.8	76.32	9,066,710
Liechtenstein	W	20,933	87	0.42	2,266	102,174	43.7	67.23	38,387
Estonia	N+	609,233	2,790	0.46	2,111	3,606,878	41.5	63.93	1,321,910
Sweden	N+	2,626,686	21,002	0.8	2,055	19,133,584	39.5	72.33	10,218,971
Andorra	S	46,824	156	0.33	2,014	249,838	42.5	66.99	77,463
Serbia	S	2,423,385	17,387	0.72	2,009	11,627,759	42.9	47.71	8,653,016
Germany	W+	36,463,485	157,791	0.43	1,881	122,332,384	44.9	76.18	83,883,596
Malta	S+	115,818	809	0.7	1,822	2,090,375	39	88.36	444,033
Luxembourg	W+	297,757	1,133	0.38	1,764	4,412,567	38.7	71.43	642,371
Switzerland	W	4,317,035	14,318	0.33	1,632	22,932,989	41.8	68.78	8,773,637
Ireland	N+	1,678,827	8,131	0.48	1,620	12,836,506	37.6	80.74	5,020,199
Monaco	W	15,442	63	0.41	1,584	78,646	54.5	70.34	39,783
Isle of Man	N	38,008	116	0.31	1,353	150,753	37.9	79.38	85,732
Netherlands	W+	8,541,997	22,909	0.27	1,331	25,984,435	41.7	68.08	17,211,447
Finland	N+	1,394,254	7,265	0.52	1,308	11,786,151	42.4	78.43	5,554,960
Denmark	N+	3,147,600	7,532	0.24	1,291	128,806,480	41.3	81.05	5,834,950
Albania	S	333,343	3,593	1.08	1,254	1,941,032	37.3	44.62	2,866,374
Channel Island	N	95,726	207	0.22	1,173	1,252,808	43.2	NA	176,463
Norway	N	1,469,061	4,325	0.29	785	11,002,430	39.3	74.61	5,511,370
Belarus	E	994,037	7,118	0.72	755	13,646,641	40.2	67.08	9,432,800
Iceland	N	207,171	219	0.11	634	1,996,384	35.9	77.82	345,393
Faeroe Island	N	34,658	28	0.08	569	778,000	37.9	76.99	49,233
Vatican City	S	29	0	0	0	NA	41.8	NA	799

On the other hand, the number of cases and the number of deaths were significantly proportional to the number of tests performed, as shown in Table 6. The CFRs were negatively correlated to the number of cases per million, the tests per population, and the rate of vaccination coverage but positively correlated to the number of deaths per million. The vaccination coverage rate was directly proportional to the number of cases per million and the number of tests per population but negatively correlated to the number of deaths per million. Europe is the continent with the highest median age. However, the correlation test of the median age with other variables was insignificant.

**Table 6:** Correlation tests of study variables across Europe. **Correlation is significant at the 0.01 level (2-tailed). *Correlation is significant at the 0.05 level (2-tailed). ^#^CFR: case-fatality rate; C/M: the number of cases per million; D/M: the number of deaths per million; Vac.: vaccination coverage rate (the percentage of people who complete the initial protocol).

		Median age	No. of cases	No. of deaths	CFRs^#^	C/M^#^	D/M^#^	No. of tests	Tests/pop.	Vac.^#^
Median age	Pearson correlation	1	0.107	-0.009-	-0.116-	0.236	0.155	0.053	-0.021-	0.162
	Sig. (2-tailed)		0.475	0.950	0.437	0.110	0.297	0.723	0.886	0.283
	N	47	47	47	47	47	47	47	47	46
No. of cases	Pearson correlation	0.107	1	0.788^**^	-0.057-	0.049	-0.020-	0.729^**^	0.012	0.174
	Sig. (2-tailed)	0.475		0.000	0.699	0.742	0.896	0.000	0.933	0.246
	N	47	48	47	48	48	47	48	48	46
No. of deaths	Pearson Correlation	-0.009-	0.788^**^	1	0.205	-0.260-	0.117	0.710^**^	-0.087-	-0.039-
	Sig. (2-tailed)	0.950	0.000		0.168	0.078	0.434	0.000	0.562	0.796
	N	47	47	47	47	47	47	47	47	46
CFRs	Pearson correlation	-0.116-	-0.057-	0.205	1	-0.623-^**^	0.719^**^	-0.045-	-0.349-^*^	-0.724-^**^
	Sig. (2-tailed)	0.437	0.699	0.168		0.000	0.000	0.761	0.015	0.000
	N	47	48	47	48	48	47	48	48	46
C/M	Pearson correlation	0.236	0.049	-0.260-	-0.623-^**^	1	-0.239-	0.014	0.551^**^	0.572^**^
	Sig. (2-tailed)	0.110	0.742	0.078	0.000		0.105	0.927	0.000	0.000
	N	47	48	47	48	48	47	48	48	46
D/M	Pearson correlation	0.155	-0.020-	0.117	0.719^**^	-0.239-	1	0.007	-0.204-	-0.455-^**^
	Sig. (2-tailed)	0.297	0.896	0.434	0.000	0.105		0.964	0.170	0.001
	N	47	47	47	47	47	47	47	47	46
No. of tests	Pearson correlation	0.053	0.729^**^	0.710^**^	-0.045-	0.014	0.007	1	0.319^*^	0.228
	Sig. (2-tailed)	0.723	0.000	0.000	0.761	0.927	0.964		0.027	0.128
	N	47	48	47	48	48	47	48	48	46
Vac.	Pearson correlation	0.162	0.174	-0.039-	-0.724-^**^	0.572^**^	-0.455-^**^	0.228	0.528^**^	1
	Sig. (2-tailed)	0.283	0.246	0.796	0.000	0.000	0.001	0.128	0.000	
	N	46	46	46	46	46	46	46	46	46

## Discussion

This study described the COVID-19 prevalence in Europe approximately three years after the emergence of SARS-CoV-2 and compared the findings globally. Europe was the continent most affected based on the absolute numbers: the number of cases and deaths. By the end of 2020, Belgium was the worst country in the world (after San Marino) based on the number of deaths per million; it has about 1,674 deaths per million [17,18]. However, significant variability in statistics happened over time. By the end of November 2022, Belgium became the twenty-eighth worst country in the world according to the number of deaths per million, while 13 European countries were among the worst 15 countries, most of them from Eastern and Southern Europe. The United States (US) was the worst affected country based on absolute numbers and the most tested. In terms of the number of deaths per million, the US was in the 16th rank, with approximately 3302 deaths per million. While Peru, a country in South America, became the worst country, it registered 6454 deaths per million [6].

In addition to the number of deaths per million, the CFR (also called infection-fatality rate) is used to assess emerging infection mortality. In the current pandemic, the CFR has been commonly used. However, unlike the mortality rate, CFR provides a short view because it does not consider the entire population. It should be understood that the information on the number of cases and deaths anywhere is based on positive tests and not on everyone who is actually infected [5]. Globally, the CFR ranged from zero to 18.07%. Across Europe, the highest CFR was 4.04%. Furthermore, the CFR varies significantly over time [17]. In France, by July 12, 2020, about 1.4 million tests were performed, and there were nearly 170,000 cases and about 30,000 deaths. Therefore, the CFR was very high (17.50%), not because the disease was serious but because the tests were limited to severe cases, i.e., mild cases were ignored (selection bias). Later, the cases increased in France due to the significant increase in the number of tests carried out; thus, the CFR decreased. Specifically, by the end of November 2022, France had performed more than 271 million tests (4.14 tests/population) slightly. So, the outcomes based on the CFR should be interpreted cautiously. In contrast, in their study, which described the COVID-19 prevalence in Europe, Vicente and Suleman claimed that the CFR is a more reliable metric than the number of deaths per million [18]. This claim contradicts what many studies have concluded [5,7,15-17,19]. One of the limitations of their study is that it included only 30 countries (including all 27 EU states). Furthermore, CFR is affected by the number of cases detected, which in turn is affected by the number of tests conducted, as shown in the example of France. Globally, some countries have a high CFR but a lower mortality rate, such as Yemen and Syria [19]. Moreover, it is crucial to consider the natural immunity acquired over time [20]. So, in epidemics, initial CFRs usually start high and trend downwards.

As of December 1, 2022, at least 6.84 billion COVID-19 tests have been performed worldwide. The largest share of tests was carried out in Europe (40.9%), while fewer tests were conducted in Africa (1.6%), and therefore, fewer cases and deaths were recorded. Detecting cases of COVID-19 and thus attributing deaths to SARS-CoV-2 depends on the availability of testing. Cases cannot be confirmed without testing, although some countries, such as North Korea, have relied on clinical recording methods [21]. Statistically, the number of deaths and cases is positively proportional to the number of tests performed, consistent with other studies [7,15,17,19]. Most European countries have performed tests more than their population. Specifically, 43 of the 47 countries (after excluding Vatican City, where there is no data on the number of tests) have performed tests more than their population. Moreover, 26 countries have tested more than twice their population. The same was almost observed worldwide, as 103 countries have performed more tests than their population [6]. In contrast, Reunion was the only African country that conducted tests more than its population [7].

We should look at the global testing strategy to understand the reasons for the huge number of tests performed. Besides the diagnostic testing strategy, screening asymptomatic people who do not have known, suspected, or reported exposure to SARS-CoV-2 is another testing strategy followed globally to control the community spread of the virus [22]. In the current pandemic, mass screening was suggested as a critical component to fight against SARS-CoV-2 and return to normalcy [23]. Therefore, mass testing campaigns were conducted in many countries. However, some world countries cannot follow the strategy of mass screening; countries differ in terms of the capacity for testing and testing strategy [7,19]. In general, high-income countries tend to perform more tests. European countries have followed a similar policy.

For global comparison, the mean deaths per million, the mean cases per million, and the mean tests per population for European countries were higher than those of the world's countries, although they recorded a lower mean CFR. Alternately, European countries, in aggregate, recorded the highest number of cases, deaths, and cases per million among the continents. However, COVID-19 statistics have been somewhat inconsistent across Europe. Northern Europe had the most significant number of tests per population and thus had a higher number of cases per million. In contrast, Eastern Europe had the least number of tests per population and thus had fewer cases per million. Based on the number of deaths per million, Western Europe was the least affected, followed by Northern Europe. Statistically, a significant difference was noticed between Eastern Europe and other parts of Europe. Also, there was a significant difference in the mean deaths per million between Northern Europe and Southern Europe. Eastern Europe was the worst based on the number of deaths per million. It should be noted that healthcare disparities across Europe possibly play a role, as Eastern Europe’s healthcare systems as a whole are well-documented to be suffering from health system defects compared to the other parts [24]. On the other hand, there was no significant difference in statistics between the EU states and non-EU countries. The reason may be that the EU countries include countries from all four parts of the continent.

Europe has the highest median age among the continents, but there was no significant difference across Europe. In contrast, the median age in Africa is 18.6 [7,9], which is the lowest. Africa has a lower median age and fewer long-term facility care than Europe. These factors partly contribute to the low burden of the COVID-19 pandemic in Africa. However, the situation is more complex because of the interaction of many factors. Unlike Europe, Africa suffers from a shortage of trained staff required for diagnostics and intensive care units (ICU), inadequate ventilators and ICU facilities (required in critical cases), lack of personal protective equipment for healthcare workers, and scarcity of funds for the health sector, as well as the high prevalence of malnutrition and other endemic diseases [25]. Additionally, Africa has less vaccination coverage rate than Europe [10]. To illustrate, by the end of 2021, about 48.85% of the world population had received two doses of the COVID-19 vaccine, while only 8.77% of people in Africa had been fully vaccinated. These factors are assumed to increase the burden of disease in Africa. Despite all this, the continent of Africa has maintained its advantage over the continents since the beginning of the pandemic [7]. However, the low COVID-19 prevalence in Africa might not reflect reality as many infected people pass without any laboratory confirmations [26]. A cross-sectional household study in Zambia reported much higher infections than reported via the limited normative testing, which showed only one confirmed case reported for every 92 community infections [27]. Anyhow, these inconsistent findings suggest considerable uncertainty over the exact COVID-19 statistics over the world. The most important reason for this variability is the difference between countries in terms of the definition of suspected cases, testing strategies, the criteria for when a result is considered positive, the capacity to perform testing, reporting methods, and other undetermined factors [7,8].

The burden of the COVID-19 pandemic has varied over the three years. In 2020, the world witnessed 1.8 million deaths attributed to COVID-19, while the second year was the worst as the number of cases and deaths tripled. The natural immunity acquired over time and the vaccination coverage play an important role in mitigating the virulence of the virus, as observed in 2022, in which the highest number of COVID-19 cases were recorded but without a relative increase in the number of deaths [20]. To illustrate, there were about 396 million COVID-19 cases and nearly 1.2 million deaths worldwide in the first 11 months of 2022, representing 55.53% and 17.90% of all cases and deaths since the start of recording COVID-19 data, respectively. Moreover, most of these cases (73.5%) and deaths (76.2%) occurred in the first six months [6,17]. Specifically, actual cases recorded in Europe are roughly equivalent to one-third of the population. In addition, the vaccination coverage rate in Europe was reasonable; about 66.8% of people have been fully vaccinated. However, as with other viruses, the SARS-CoV-2 virus is subject to continuous mutations, and thus new variants arise [28]. So, it is expected that more cases of COVID-19 will be reported in the cold season (e.g., December to March) if testing continues, while other viruses will be neglected [29]. In contrast, if a country's health authorities choose not to test for COVID-19, such cases will not be known. Still, they will be classified under other respiratory diseases if patients consult health providers.

Therefore, unless the end of the pandemic is announced, it is crucial to reconsider the definition of a suspected case of COVID-19 and set more specific criteria, especially since it is estimated that at least 6.6 billion infections with SARS-CoV-2 have occurred worldwide [30]. Because pneumonia is considered the most severe frequent manifestation of infection with SARS-CoV-2 [1], it might be appropriate that the criteria of the suspect case of SARS-CoV-2 infection should include lower respiratory symptoms, such as dyspnea, with abnormal vital signs and chest X-ray abnormalities suggestive of pneumonia, as well as a history of contact with a probable or confirmed case of COVID-19, not only upper respiratory tract symptoms.

Finally, this study provides an updated overview of COVID-19 statistics around Europe and how much they differ across Europe. However, there are some limitations to this study. The most important limit is that the quality of information obtained depends on the raw data quality. Not all countries were reporting COVID-19 statistics at the same frequency and quality. Moreover, most COVID-19 data were obtained from a single source.

## Conclusions

By the end of November 2022, Europe was the continent with the most cases of COVID-19, the most deaths, and the most tests performed, even though it accounts for 9.4% of the world’s population. However, COVID-19 data were somewhat inconsistent across the four parts of Europe regarding the mean CFR, the mean cases per million, the mean deaths per million, and the mean vaccination coverage rate. A significant difference was noticed between Eastern Europe and others, especially Northern Europe and Western Europe. The COVID-19 statistics were almost similar between the EU states and non-EU states. Given the natural immunity acquired during the three years and the excellent vaccination coverage in Europe, it is essential to reconsider the definition of a suspected case and establish more specific criteria for testing.
